# Caveolin-1 function at the plasma membrane and in intracellular compartments in cancer

**DOI:** 10.1007/s10555-020-09890-x

**Published:** 2020-05-27

**Authors:** L. Simón, A. Campos, L. Leyton, A. F. G. Quest

**Affiliations:** 1 Laboratory of Cellular Communication, Center for studies on Exercise, Metabolism and Cancer (CEMC), Programa de Biología Celular y Molecular, Facultad de Medicina, Universidad de Chile, Santiago, Chile; 2 Advanced Center for Chronic Diseases (ACCDIS), Santiago, Chile

**Keywords:** Caveolin-1, Localization, Organelles, Metastasis

## Abstract

Caveolin-1 (CAV1) is commonly considered to function as a cell surface protein, for instance in the genesis of caveolae. Nonetheless, it is also present in many intracellular organelles and compartments. The contributions of these intracellular pools to CAV1 function are generally less well understood, and this is also the case in the context of cancer. This review will summarize literature available on the role of CAV1 in cancer, highlighting particularly our understanding of the canonical (CAV1 in the plasma membrane) and non-canonical pathways (CAV1 in organelles and exosomes) linked to the dual role of the protein as a tumor suppressor and promoter of metastasis. With this in mind, we will focus on recently emerging concepts linking CAV1 function to the regulation of intracellular organelle communication within the same cell where CAV1 is expressed. However, we now know that CAV1 can be released from cells in exosomes and generate systemic effects. Thus, we will also elaborate on how CAV1 participates in intracellular communication between organelles as well as signaling between cells (non-canonical pathways) in cancer.

## Introduction

Caveolin-1 (CAV1) is a small, oligomeric scaffolding protein, typically required to generate membrane curvature in structures, such as caveolae [123, 174]. Moreover, CAV1 binds to many other proteins, controls cholesterol homeostasis, and regulates a variety of cell functions, such as endocytosis, receptor internalization, cholesterol accumulation, and cell signaling, proliferation, and death [29, 123]. CAV1-mediated control of signaling events is relevant in cancer. As an example, may it suffice to mention the interaction between CAV1 and Rho GTPases, such as RhoC, which favors the development of metastasis by stimulating α5-integrin expression and Src kinase-dependent activation of the p130Cas/Rac1, FAK/Pyk2, and Ras/Erk1/2 pathways [6, 115]. Importantly, however, CAV1 also functions as a tumor suppressor by aiding E-cadherin in the sequestration of β-catenin, thereby impeding activation of the β-catenin/Tcf-Lef-dependent transcription of genes, like survivin, cyclooxygenase-2, cyclin D1, and many others that favor cancer development [140, 165, 166]. Thus, CAV1 participates both as a tumor suppressor and promoter in cancer (reviewed in [52, 123, 132]).

CAV1 is synthesized, then oligomerizes, and is inserted into the ER membrane through the classical membrane/secretory protein translocation pathway. The hydrophobic domain serves as an ER membrane anchor and adopts a loop configuration exposing the N- and C-terminal domains to the cytoplasm [109]. In addition, the N-terminal domain has a DXE (Asp-X-Glu) sequence that allows CAV1 to concentrate in ER exit sites and then be transported to the Golgi apparatus with the help of the coat protein II (COPII) machinery. Once in the Golgi apparatus, CAV1 undergoes conformational changes and assembles (in a cholesterol-dependent process) into larger and more stable complexes of about 160 caveolin molecules, lipids, and membrane raft-associated cargos [59]. These are transported to the plasma membrane in vesicles and inserted as planar caveolar domains, into which PTRF/cavin1 can be recruited, as well as other cavins, so as to generate the caveola structures [59, 161]. Thus, because the protein traffics to the cell surface and can then be internalized, CAV1 is also detectable at many intracellular sites. While subcellular location is often considered fortuitous, it is becoming increasingly clear that functionally important organelle-associated pools exist, although their precise role remains largely obscure [44].

In caveolae or membrane rafts at the plasma membrane, CAV1 may serve to generate signaling hubs that control many downstream events. As mentioned, CAV1 aids, together with E-cadherin, in sequestering β-catenin to the plasma membrane and in doing so regulates β-catenin/Tcf-Lef-dependent signaling [45, 165, 166]. In addition, CAV1 is also implicated in the internalization and turnover of the transforming growth factor beta (TGF-β) receptor [36]. Some of these roles are related to CAV1 function as a scaffolding protein; nevertheless, alternative possibilities exist. For instance, Raf-1 is a proto-oncogene and serine/threonine protein kinase, which is recruited to caveolae after epidermal growth factor (EGF) stimulation and its presence there is necessary for the activation of the MAP kinase pathway [107]. However, others suggest that caveolae are depleted of membrane proteins [155] and participate indirectly in, for instance, Ras-dependent signaling by changing lipid organization and cholesterol content in domains of the plasma membrane [4, 144]. Thus, CAV1 plays a significant role in modulating cell signaling events at the cell surface but can do so in different ways.

As indicated, CAV1 has been reported to function both as a tumor suppressor and as a promoter of tumor progression and metastasis (reviewed in [24, 133, 147]). A large number of reports support the role of CAV1 as a tumor suppressor, associating the reduced expression of CAV1 with cell transformation [12, 173, 178]. On the other hand, the re-expression of CAV1, often observed in later stages of cancer, has been linked to tumor progression, multi-drug resistance, and metastasis [38, 73, 89, 95]. To what extent such variability may be linked to the different ways in which CAV1 modulates signaling at the cell surface or elsewhere is currently an area of great interest.

The central region of CAV1 (residues 82–101), referred to as the caveolin scaffolding domain (CSD), is proposed to bind to many other proteins (containing a region enriched in aromatic residues termed caveolin-binding motif, CBM) and prevent downstream signaling events [118]. For instance, CAV1 reportedly interacts with G proteins and suppresses their basal activity by inhibiting GDP/GTP exchange [85, 124]. Moreover, this domain is implicated in regulating cell proliferation and survival by inhibiting cell signaling proteins, such as eNOS, Gi2α, and PKCα [136]. Furthermore, the CSD also regulates Ca^2+^ influx into cells by interacting with and modulating the activity of certain ion channels, such as TRPC1 [117, 160], thereby leading to the alteration of cellular processes, including cell proliferation [25] and tumor invasion [117]. Moreover, this domain is suggested to be crucial in controlling cell migration, possibly *via* STAT3, and also cell cycle progression in several cancer cell lines [117].

In striking contrast, other reports describe how this domain reduces the activity of the serine/threonine protein phosphatases PP1 and PP2A in order to sustain Akt activation and thereby promotes cell survival in prostate [83] and pancreatic cancer [61]. Others implicate the CSD in mediating interactions between CAV1 and Rho GTPases. For instance, Rufini and colleagues showed that the disruption of this interaction by overexpressing CSD peptides in metastatic melanoma cells led to diminished survival and extravasation of these cells [6]. Additionally, Hordijk and collaborators showed that CAV1 promotes directional cell migration *via* CSD-mediated interaction with the C-terminal domain of Rac1 [114].

However, several reports suggest that CAV1 effects are not due to the interaction between a possible CBM in target proteins and the CSD in CAV1. In general, proteins associated with caveolae are not enriched in CBM. Moreover, CBM are hydrophobic segments enriched in aromatic residues that are buried within the protein structure, making it difficult to envision their participation in the interaction with other proteins [20, 33]. In addition, the CSD is buried in the membrane and inaccessible for the interaction with cytoplasmic proteins [5]. This data points towards the relevance of other mechanisms through which CAV1 regulates signaling events, such as those previously mentioned involving alterations in the lipid domain organization or *via* interactions mediated by segments of the protein that are phosphorylated.

In this respect, CAV1 phosphorylation on tyrosine 14 (pY14-CAV1) [105] has emerged as being important. Phosphorylation at this site is associated with modulation of focal adhesion dynamics, cell migration, invasion, and tumor metastasis as will be detailed later on [48, 71, 115, 119]. Interestingly, Nabi and colleagues showed that CAV1 phosphorylation on tyrosine 14 stabilizes focal adhesion proteins and promotes cell motility in a CSD-dependent manner in prostate cancer cells [105]. The authors suggest that CAV1 phosphorylation favors the interaction between the CSD and several focal adhesion proteins, which in turn promotes focal adhesion tension and cell migration, thus identifying pY14-CAV1 as a molecular regulator of such processes (see also review in this same special edition by Nabi and co-workers). Other reports also mention the existence of a link between pY14-CAV1 and the CSD, whereby CAV1 phosphorylation on tyrosine 14 is suggested to trigger conformational changes within the oligomeric structure of CAV1 molecules that increase CSD accessibility for interactions with other proteins [150, 186].

**Fig. 1:**
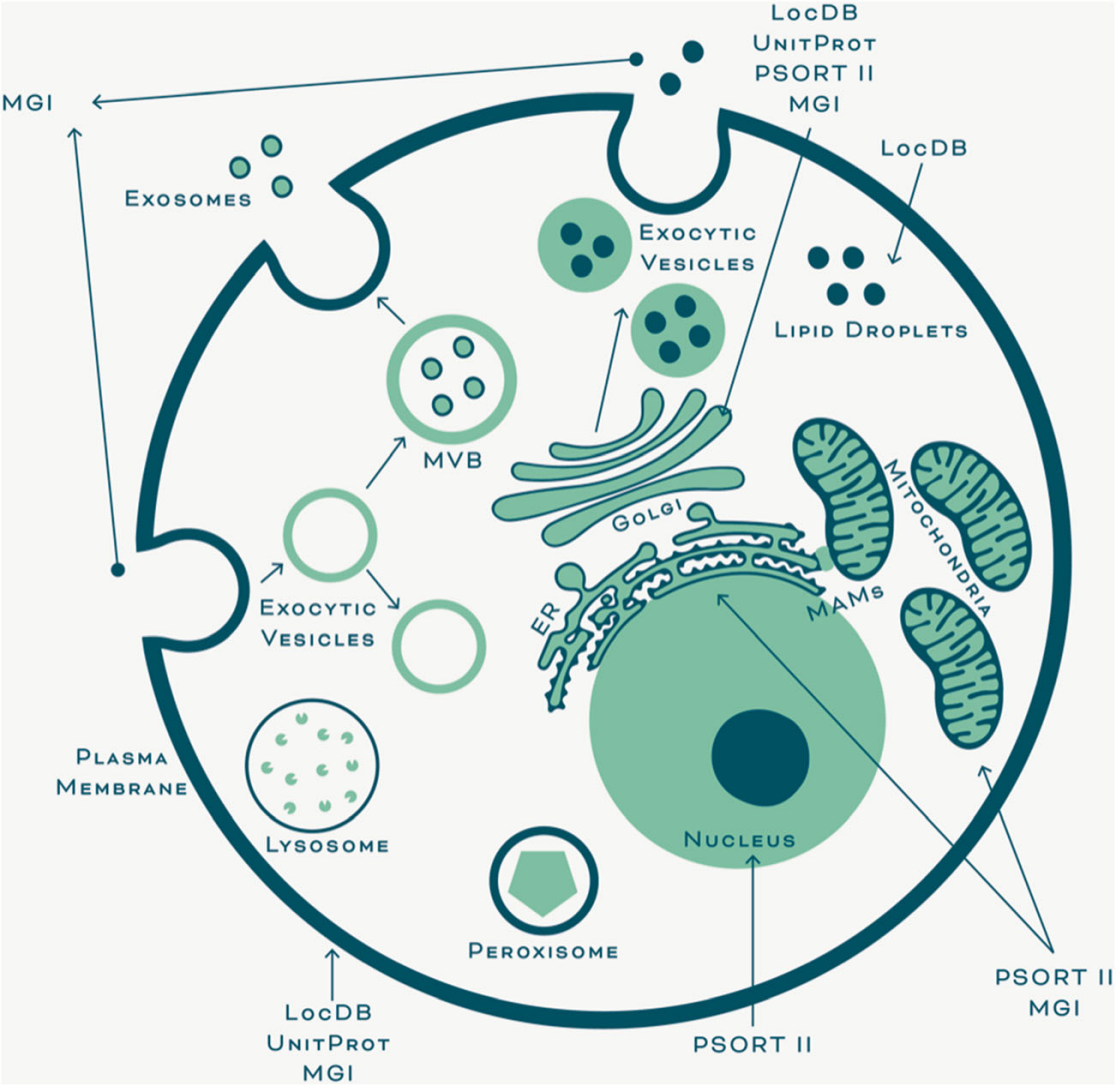
Subcellular localizations of CAV1. Representative scheme that summarizes the subcellular compartments where CAV1 is suggested to be localized as supported by the UniProt, PSORT II, MGI, and LocDB databases and select references (see Table 1). According to the data, CAV1 is present in the plasma membrane, Golgi apparatus, ER, nucleus, endocytic as well as exocytic vesicles, multivesicular bodies (MVB), and lipid droplets. Additional evidence places CAV1 at mitochondria-ER interphase sites (localizing CAV1 indirectly to mitochondria), referred to as mitochondria-associated ER membranes (MAMs), as well as lysosomal, peroxisomal, and exosomal membranes, as will be discussed in this review

The regulation of all the signaling pathways described above is attributed to CAV1 presence at the plasma membrane (canonical and predominantly studied roles). However, an analysis of information available in data bases reveals that CAV1 localizes to many cell organelles and subcellular compartments (Table 1 and Fig. 1), suggesting that CAV1 function is not likely to be limited to the plasma membrane, and moreover, that these CAV1 pools modulate important cellular functions that in general are less characterized (non-canonical roles).

**Table 1:** Subcellular localizations of CAV1. The localizations indicated here are inferred from protein topology analysis using UniProt knowledgebase, sequence-based predictions using PSORT (Prediction of Protein Sorting Signals and Localization Sites in Amino Acid Sequences), gene ontology obtained from the Mouse Genome Informatics (MGI) database as well as experimental results obtained from the Protein Localization Database (LocDB) and the indicated references

Localization	UniProt	PSORT II	MGI	LocDB	
	Topology	Prediction	Gene ontology	Experimental	
Golgi apparatus	+	+	+	+	Fridolfsson et al. [44]
Plasma membrane	+		+	+	Fridolfsson et al. [44]
Endoplasmic reticulum		+	+		Fridolfsson et al. [44] Hayer et al. [59]
Cytosol		+	+		
Mitochondria		+	+		Fridolfsson et al. [44]
Nucleus		+			Fridolfsson et al. [44]
Endosome			+		Fridolfsson et al. [44]
Non-membrane-bounded organelle			+		
Cell projection			+		Joshi et al. [71]
Cytoplasmic vesicle			+		Fridolfsson et al. [44]
Lipid particle				+	Fridolfsson et al. [44]

Thus, here, we will review in detail the literature available on the role of CAV1 in cancer and specifically the canonical and non-canonical functions of the proteins associated with CAV1 at the plasma membrane and in other subcellular localizations, respectively.

## Canonical role of CAV1 at the plasma membrane

### CAV1 as a tumor suppressor or promoter

At early stages, CAV1 has been ascribed roles as a tumor suppressor in colorectal, breast, lung, and liver cancers. For instance, the reduction of stromal CAV1 is associated with poor patient survival in breast cancer [182]. CAV1 downregulation promotes proliferation, while the overexpression induces apoptosis in lung cancer cell lines [49]. In addition, CAV1 downregulation inhibits senescence, while promoting lung tumor development and increasing mortality in mice [173].

The regulation of CAV1 protein levels is poorly understood. Low and Nicholson reviewed the effect of epigenetic regulation of CAV1, concluding that hypermethylation of the CAV1 promoter decreases protein levels in breast and prostate cancer [98]. This result is also observed in gastric cardia adenocarcinoma and alveolar rhabdomyosarcoma, where mRNA and protein levels are downregulated by hypermethylation of CpG islands, which is associated with a decrease in patient survival [51, 67]. Treatment with DNA-hypomethylating agents, such as 5-aza-2′-deoxycytidine, restores the expression of CAV1, not only in breast and prostate but also in ovarian, colon, and liver cancer cells [98]. In addition, treatment of colon cancer cells with histone deacetylase inhibitors, such as trichostatin A, increases CAV1 expression and prevents cell proliferation [35]. Besides, epigenetic regulation of CAV1-associated proteins is involved in cancer development. For instance, hypermethylation of the PTRF/cavin1 promoter leads to a reduction in caveola formation and Ewing sarcoma development. The re-introduction of PTRF/cavin1 and CAV1 increases caveola number, promotes cell death, and decreases tumor size [68].

On the other hand, the analysis of CAV1 expression in samples of patients at early stages of colorectal cancer reveals that protein levels are downregulated, but there is no correlation with a reduction in mRNA levels, probably due to post-transcriptional regulation involving miRNA 124 [164].

In terms of the signaling pathways implicated in CAV1-associated tumor suppression, there are several studies on its role as a plasma membrane-bounded protein (see Section 1). Furthermore, CAV1 has been shown to control proliferation and apoptosis of human lung carcinoma cell lines. In these cells, CAV1 reduces cadherin-11/Stat3/Rac1 signaling by interactions at the plasma membrane involving the scaffolding domain [49]. Also, CAV1 stabilizes cell–cell contacts and inhibits the spread of tumor cells during metastasis by favoring the plasma membrane localization of E-cadherin and p120-Catenin and the development of adherent junctions in ovarian carcinoma cell lines [108]. Alternatively, EGF treatment induces caveola-dependent endocytosis of plasma membrane E-cadherin/β-catenin complex, disrupts cell–cell contacts, downregulates E-cadherin as well as CAV1, and thereby promotes invasion of cancer cells [99].

Moreover, CAV1 has been proposed to inhibit TGF-β signaling, *via* caveolae/membrane raft-mediated endocytosis and lysosomal degradation of TGF-β receptors [28, 175]. On the one hand, CAV1 interacts with TGF-β receptors, abolishing their interaction with SMAD proteins and downstream events [28]. On the other hand, caveolae and membrane rafts create a favorable environment for the recruitment of ubiquitin ligases which promote the ubiquitination and degradation of CAV1 and TGF-β receptors [28]. In this case, CAV1 recruits ubiquitinated TGF-β receptors in the plasma membrane and aids in transport to lysosomes, thereby inhibiting TGF-β signaling. The reduction of the ubiquitination of CAV1 and TGF-β receptors by the deubiquitinase POH1 reduces lysosomal degradation of the TGF-β receptors in liver cancer cells. In doing so, POH1 promotes migration and invasion *via* TGF-β signaling [175].

Quite to the contrary, CAV1 promotes metastasis and multi-drug resistance in late stages of cancer. The expression of CAV1 is higher in cancer compared to benign tissue and correlates with poor prognosis [127]. The overexpression of CAV1 is associated with increased cell survival, anchorage-independent growth, and epithelial-mesenchymal transition, migration, invasion, and resistance to anti-neoplastic drugs [38, 94, 111, 127, 176].

An important step during the metastatic process is the epithelial-mesenchymal transition (EMT), where CAV1, E-cadherin, and the TGF-β receptor at the plasma membrane are implicated [94, 127]. Looi and collaborators reviewed the role of E-cadherin in EMT, where the protein is replaced by N-cadherin, which disrupts adherent junctions and promotes cancer cell spread [97]. Interestingly, during tumor progression, the switching of cadherins at the plasma membrane is associated with augmented CAV1 expression. In this sense, CAV1 increases migration of melanoma metastatic cells, but the co-expression of CAV1 and E-cadherin inhibits tumor formation and lung metastasis [94]. In addition, CAV1 reprograms the TGF-β signaling pathway from suppressing tumor formation to being oncogenic. The lack of CAV1 increases the expression of epithelial, but reduces the expression of mesenchymal TGF-β target genes in metastatic prostate cancer cells. Moreover, CAV1 silencing increases the expression of E-cadherin, integrin β4, and desmoplakin, thereby favoring epithelial integrity and inhibiting the tumor spread [127].

Recent studies from our laboratory indicate that not only the expression of CAV1 at the plasma membrane but also the phosphorylation of CAV1 on tyrosine 14 is necessary to increase the metastatic potential of cancer cells *in vitro* and *in vivo* [119]. Src family kinases are responsible for CAV1 phosphorylation on tyrosine 14 and alternatively, phosphorylated CAV1 aids in the recruitment of particularly Src to the plasma membrane, leading to accumulation and activation of Src in focal adhesions [50]. In metastatic gastric cancer cells, CAV1 promotes resistance to anoikis in a Src-dependent manner by activation of the EGFR/integrin βI, PI3K/pAkt, and MEK/ERK signaling pathways [176]. In addition, in hepatocellular cancer cell lines, CAV1 is required for the induction of survival *via* the TGF-β/EGFR/pAkt signaling pathway [111].

### Role of CAV1 in migration

The presence of CAV1 at the plasma membrane is necessary for migration and invasion processes, since disruption of membrane rafts by targeting CAV1 or cholesterol reduces the metastatic potential of cancer cells [53, 181]. Also, the phosphorylation of CAV1 is required for melanoma metastasis. The expression of non-phosphorylatable CAV1 abolishes the migration, invasion, and metastasis. On the other hand, the expression of wild-type and phosphomimetic CAV1 increases the metastatic potential of these cells [119]. Although CAV1 inhibits migration through its scaffolding domain in HeLa cells [117], CAV1 increases migration *via* several signaling pathways in metastatic cancer cells [15, 37, 119, 121].

On the one hand, pY14-CAV1 acts as a stabilizer of focal adhesions through its scaffolding domain in metastatic cells. For instance, pY14-CAV1 binds focal adhesion kinase (FAK) and vinculin at the plasma membrane level promotes focal adhesion traction and migration as mentioned earlier on [105]. Furthermore, pY14-CAV1 promotes focal adhesion turnover during migration [119, 168].

On the other hand, CAV1 enhances migration *via* activation of the Rab5/Rac1 pathway. CAV1 recruits p85α (a Rab5 GTPase-activating protein), thereby preventing Rab5 inactivation. Active Rab5 increases the recruitment of Tiam1 (a Rac1 guanine nucleotide exchange factor) to early endosomes, allowing the activation of Rac1 [37]. In addition, the presence of pY14-CAV1 in membrane rafts is required for mechanical stress-induced metastasis mediated by PI3K(p85)/Akt/mTOR activation. In this case, mechanical stress induces cell motility, invadopodia formation, gelatin degradation, cytoskeleton remodeling, and metastasis, which are all blocked by the treatment with membrane raft disruptors, such as methyl-β-cyclodextrin [181].

Moreover, desmoglein 2 and CAV1 play an important role in the migration of squamous carcinoma cells. Desmoglein 2 removes CAV1 from the cell surface and disrupts membrane rafts. In this way, desmoglein 2 activates the EGFR signaling pathway and increases migration in a Src-dependent manner [121]. In addition, in metastatic cancer cells, activation of the EGFR promotes the integrin/galectin3/pY14-CAV1/RhoA signaling pathway, which stimulates cytoskeleton remodeling and cell migration in a Src-dependent manner [15].

Interestingly, CAV1 presence and function at the plasma membrane are not only related to caveolae. Historically, CAV1 has been associated with its capacity to recruit and retain proteins within caveolae. Nevertheless, more recent studies reported on the exclusion of transmembrane proteins from caveolae [155], suggesting that CAV1 modulates functions in caveolae and also other compartments within the plasma membrane. In fact, non-caveolar CAV1 oligomers, referred to as CAV1 scaffolds, participate in EGFR signaling, endocytosis, and focal adhesion dynamics [78]. The role of caveolar and non-caveolar CAV1 have also been studied in PC3 prostate cancer cells, which express high levels of CAV1, but lack of PTRF/cavin1 [63]. In this cell line, the caveola formation observed upon PTRF/cavin1 overexpression correlates with a reduction in migration that is associated with CAV1 accumulation in caveolae at the cell rear, increases in E-cadherin, and decreases in vimentin and metalloprotease 9 together with Rac-1 depolarization, all processes related to the inhibition of EMT. On the other hand, the absence of PTRF/cavin1 permits the polarized accumulation of non-caveolar CAV1 and Rac1 in leading edge protrusions and increases migration. All these observations confirm the notion that CAV1 has different functions according to its localization, even within the plasma membrane [8, 64]. Of note, caveola formation or stability is assisted by other proteins implicated in cancer, such as PACSINs [106, 146] and EHDs [84, 110]. A detailed discussion of these proteins and their participation in related processes is however beyond the scope of this review and can be found elsewhere [58, 66, 91, 131, 149].

In summary, CAV1 at the plasma membrane participates in a large number of signaling events that contribute to its role both as a tumor suppressor and promoter of metastasis. How these vastly distinct roles are coordinated within seemingly similar sites remains an enigma that merits resolving in the future.

## Non-canonical role of CAV1 in mitochondria

In general, cancer cells utilize aerobic glycolysis instead of mitochondrial oxidative phosphorylation, which is a metabolic event known as the Warburg effect [177]. Although aerobic glycolysis is an inefficient way of producing ATP, it is very effective in generating the metabolites required for cell proliferation, migration, and invasion [56, 57, 172]. Most recent studies reported on the existence of different tumor microenvironments with symbiosis between different types of cancer cells. Hypoxic cancer cells breakdown glucose by glycolysis to produce lactate, which is used by oxygenated cancer cells *via* mitochondrial respiration to generate energy [158]. Moreover, cancer cells reprogram stromal cells to provide metabolites required to meet their anabolic demands [100].

In addition, there are several reports which explain the possible reasons why the loss of CAV1 in tumor stroma is considered a poor prognostic marker in cancer from a metabolic point of view. In this regard, Lisanti and colleagues initially described that fibroblasts obtained from CAV1-deficient mice and CAV1-deficient tumor stroma samples from human breast cancer patients display catabolic features with a shift towards aerobic glycolysis and autophagy/mitophagy due to augmented oxidative stress [13, 125, 179]. These results led the authors to propose a two compartment model of tumor metabolism, known as the “reverse Warburg effect” which proposes that a glycolytic, CAV1-deficient tumor stroma may transfer catabolites, such as ketones, l-lactate, fatty acids, and amino acids, to anabolic tumor cells and thus stimulate mitochondrial metabolism in cancer cells [179]. However, to the contrary, CAV1 knockdown has also been described to decrease aerobic glycolysis, which was mainly reflected in diminished lactate accumulation and intracellular ATP levels accompanied by increased autophagy *via* AMPK-p53 signaling in colon cancer cells [55, 116]. Thus, CAV1 appears to participate in molecular mechanisms that control metabolic switching both in the stromal and cancer cell compartments.

### CAV1 localization in mitochondria-associated membranes

In terms of mitochondrial localization of caveolin proteins, some reports indicate that CAV3 in particular, is transferred to this organelle as a consequence of interactions between mitochondria and caveolae in response to sublethal ischemia in cardiac myocytes [43, 44]. As for CAV1, there are several reports which have detected this protein not only at the mitochondrial level [86], but rather enriched in mitochondria-associated ER membranes (MAMs) [19, 143]. MAMs are contact sites between the ER and mitochondria important for Ca^2+^ and lipid homeostasis [135, 143], which when purified contain proteins from the outer mitochondrial membrane and inter-membrane mitochondrial space, but not from the inner mitochondrial membrane [143]. ER and mitochondria interact directly allowing for mitochondrial Ca^2+^ uptake through at least three multiprotein complexes at the ER surface (IP_3_R), outer mitochondrial membrane (VDAC1), and inner mitochondrial membrane (MCU) [18, 41, 139]. Ca^2+^ stimulates pyruvate dehydrogenase and the F0/F1-ATPase required for pyruvate decarboxylation, Krebs cycle activity, and ATP synthesis [26, 163], thus leading to the suggestion that mitochondrial Ca^2+^ uptake promotes mitochondrial metabolism.

Numerous studies have reported on the role of CAV1 in metabolism. For instance, CAV1 null mice develop substantial metabolic alterations and mitochondrial dysfunction in white adipose tissue, associated with compensatory gluconeogenesis and reduced steatosis in the liver [7]. Another report describes that the absence of CAV1 in brown adipose tissue leads to decreased β-oxidation and substantial alterations in mitochondrial morphology, possibly due to an altered osmotic gradient occurring between the inner mitochondrial membrane and the cytoplasm of these adipocytes after being exposed to fasting/cold treatment [32]. Normal mouse embryonic fibroblast cells derived from CAV1-knockout mice are characterized by mitochondrial dysfunction, cholesterol accumulation, and apoptosis [14]. In addition, CAV1 knockdown in mouse embryonic fibroblasts inhibits mitochondrial respiration and ATP production due to impaired cardiolipin biosynthesis and SIRT1 signaling. These cells have higher expression levels of p53 and p21 (cell cycle arrest markers) and develop premature senescence [184]. In an opposite manner, cancer cells with mitochondrially localized CAV1 are more resistant to ER stress, have a more stable mitochondrial membrane potential, and have increased mitochondrial biogenesis and cell survival [43]. Thus, CAV1 appears to be necessary for mitochondrial functionality in normal cells, but the overexpression of CAV1 in cancer cells may promote malignancy in these cells.

Moreover, the behavior of normal and cancer cells with alterations in CAV1-expression is different in MAMs. Normal cells derived from CAV1 knockout mice have less contact surface between ER and mitochondria, which leads to cholesterol accumulation in MAMs [143]. This effect was also described by Pol and collaborators, who detected mitochondrial cholesterol accumulation and concomitant mitochondrial dysfunction in normal cells derived from CAV1 knockout mice. In these cells, CAV1 knockout reduces mitochondrial metabolism, but increases reactive oxidative species production and apoptosis [14]. On the other hand, CAV1 overexpression inhibits ER-mitochondria communication and remodeling upon ER stress in cancer cells, such as HeLa cells overexpressing CAV1 or MDA-MB-231 cells with high endogenous levels of CAV1. These cells have decreased Ca^2+^ levels and mitochondrial metabolism. Dynamin-related protein 1 (DRP1) phosphorylation by PKA is required for mitochondrial stability upon ER stress. By inhibiting PKA, CAV1 reduces pDRP1 levels and thereby affects mitochondrial function [19]. One of the limitations of this study was the fact that the authors were unable to define whether the aforementioned effects of CAV1 were due to the specific localization of this protein within MAMs or outside these structures. Thus, the authors considered that CAV1 located outside the mitochondria should also be taken into account when assessing effects on mitochondrial metabolism [19].

CAV1 is considered a key regulator of mitochondrial function that is important for its role as a tumor suppressor during cancer development. For instance, in H-Ras-transformed fibroblasts, CAV1 re-expression promotes Ca^2+^ reentry, thus making them prone to cell death [137]. Indeed, any alteration to the CAV1/Ca^2+^ axis triggers mitochondrial dysfunction and apoptosis in transformed cells [137, 138]. Interestingly, however, CAV1 has also been shown to promote tumor development by regulating Ca^2+^ homeostasis in breast, stomach, lung, colon, and liver cancers [138].

## Non-canonical role of CAV1 in ER and lipid droplets

As indicated above, CAV1 is important in the formation of caveola structures at the cell surface. Initially, CAV1 is synthesized in the endoplasmic reticulum (ER), oligomerized, and transported to the Golgi complex and then on to the plasma membrane as large complexes associated with lipids and membrane raft-associated cargos *via* vesicular carriers [30, 59]. Once at the plasma membrane, CAV1 recruits PTRF/cavin1 required for caveola formation [11, 59]. In addition, CAV1 domains at the plasma membrane are enriched in phospholipids, such as phosphatidylserine and phosphatidylinositol-4,5-bisphosphate, which are necessary for cavin protein recruitment during the formation of the cave-like structures [77]. Thus, specific phospholipid composition as well as the presence of cholesterol are essential in caveola assembly and composition.

Furthermore, CAV1 aids in shuttling cholesterol between the plasma membrane and the ER/Golgi and also in the trafficking Golgi resident proteins back to the cell interior from the cell surface [44]. Additionally, CAV1 plays a role in the formation and stabilization of lipid droplets. These cytosolic storage organelles of neutral lipids are known to act as major regulators of lipid metabolism, trafficking, and signaling in several *in vitro* and *in vivo* models exposed to stress [128, 156]. Concerning lipid droplet biogenesis, CAV1 promotes accumulation of lipids and proteins at specific sites in the ER before entering these organelles [70]. Moreover, a positively charged sequence in the CSD along with the last 20 residues of CAV1 are held responsible for the sorting of CAV1 into lipid droplets [70, 74]. Simulation of lipid droplet biogenesis reveals that CAV1 reduces bilayer thickness of lipid aggregates and thereby reduces the energy barrier to facilitate pinching-off of these aggregates from the host bilayer [129].

In the context of cancer, these anti-lipotoxic organelles tend to accumulate in tumor cells with an aggressive phenotype, as well as in tumor cells exposed to hypoxic or nutrient/lipid-deprived conditions. Furthermore, recent studies suggest that these dynamic organelles are capable of abolishing nutrient and oxidative stress and, therefore, promote tumor cell survival and growth by protecting, for instance, their contents from damage due to peroxidation [128, 156]. Moreover, while the majority of normal tissues synthesize new structural lipids by using extracellular pools of lipids, tumor cells may prefer *de novo* fatty acid synthesis, despite the availability of these extracellular lipids, in order to meet their specific lipid requirements and/or to maintain proliferation in a stressful environment [128, 141].

Human metastatic breast cancer cell lines, such as the triple negative MDA-MB-231 cells, contain greater levels of cholesterol and fatty acids in lipid droplets when compared to non-metastatic and hormone-responsive breast cancer cells, like MCF-7. Coincidentally, MDA-MB-231 cells express high endogenous levels of CAV1, LDL receptors, and acetyl-CoA:cholesterol acyltransferase 1 (ACAT1) enzymes [2, 3, 156], which facilitate incorporation into LDL particles and promote proliferation [2]. Moreover, Siddiqui and colleagues showed that the migration potential of MDA-MB-231 cells is dependent on ACAT1 and correlates with increased lipid accumulation in these cells [3]. Furthermore, treatment with ACAT1 inhibitors reduces LDL receptor expression and LDL-enhanced proliferation [3]. Another report shows that the treatment of triple negative breast cancer cells with bitter melon extract reduces the accumulation of esterified cholesterol, ACAT1, and LDL receptor expression, thereby reducing tumor growth in mammospheres implanted into mice [152]. Interestingly, ACAT1 inhibition has been reported to promote downregulation of the CAV1/MAPK pathway, which enhances pancreatic cancer aggressivity [82]. Therefore, ACAT1 appears to increase the tumor-promoting function of CAV1, by favoring LDL uptake, as reported in endothelial cells [42], and also the formation and stabilization of lipid droplets, which aid in sustaining tumor cell proliferation under adverse conditions.

Gao and collaborators also describe the adjuvant effects of simvastatin, an hydroxymethyl glutaryl CoA reductase inhibitor or statin that delays the progression of castration-resistant prostate cancer by blocking cholesterol biosynthesis and thereby regulating the expression of CAV1 [47]. Also, lovastatin, in combination with non-steroidal anti-inflammatory drugs, decreases the expression and membrane localization of CAV1. This leads to the inhibition of CAV1-dependent cell survival signals mediated by Akt activation, as well as other downstream signaling effectors like ERK and STAT3 in HCT-116 cells [54].

## Non-canonical role of CAV1 in endosomes and lysosomes

### Endocytosis and autophagy

In general terms, the process of endocytosis involves the inward budding of vesicles that transport several macromolecules from the plasma membrane into the cell [104]. This process is relevant not only to internalization *per se* but also for many other cellular functions, such as cell polarization, turnover of cell surface receptors, and cell–cell communication in response to extracellular stimuli [101, 112]. In addition, the endocytic process may lead to the formation of endocytic vesicles coated with clathrin or lacking this coat protein (clathrin-independent endocytosis). In the latter case, these vesicles are of plasma membrane or caveolar origin and are mainly composed of sphingolipids and cholesterol. Such microdomains are enriched in G protein-coupled receptors and GPI-anchored proteins involved in several signal transduction events [101, 103, 104]. In this context, the group of Sandvig studied the dynamic properties of the pinching-off or internalization of caveolae, focusing on the role of CAV1. They observed that the exchange rate of CAV1 between the plasma membrane and other intracellular pools is slow, thus implying that these bulb-shaped structures may be immobile at the cell surface and that their stability is controlled by CAV1 [170]. Nevertheless, others indicate that caveolae do not behave as immobile structures because they can efficiently bud as endocytic vesicles [16] or rapidly flatten into the plasma membrane in response to mechanical stress [157].

Several reports suggest that CAV1 not only impedes the pinching-off of caveolae from the cell surface but also negatively regulates the internalization of many proteins that are found in membrane rafts enriched with caveolae. Such is the case of the autocrine motility factor (AMF), a multifunctional protein with distinct functions depending on whether the protein is found inside (cytosolic glycolytic enzyme) or outside (extracellular cytokine) the cell [39, 113]. As a cytokine, AMF can bind to surface receptors and regulate cell motility, signal transduction, and protein ubiquitination in an autocrine manner [21]. Notably, the majority of the reports describe AMF as a secretable protein that, like its receptor (AMFR), is highly expressed in gastric, endometrial, and breast cancers, among others [65, 76, 88]. Specifically, in disease, AMF is suggested to promote tumor angiogenesis, cell migration and proliferation, as well as being anti-apoptotic [65]. In addition, decreased expression of CAV1 has been associated with increased AMF endocytosis upon NIH-3T3 cell transformation by Ras or Abl oncogenes [81]. Conversely, when non-transformed cells are transfected with AMF, the protein promotes cell transformation and survival *via* PI3K/Akt signaling, along with a decrease in CAV1 expression [167]. Thus, the diminished levels of CAV1 that are observed as a consequence of the cell signaling events triggered by AMF render CAV1 unable to control AMF turnover. Therefore, CAV1 function as a tumor suppressor appears attributable, at least in part, to its ability to counteract the function of tumor-promoting proteins like AMF.

An important trait that characterizes cancer cells is their deranged endocytosis [104]. Indeed, several membrane proteins required for endocytosis become dysfunctional during cancer development, as is the case for CAV1. For instance, CAV1 and dynamin-2, which are both important for caveola-mediated endocytosis and caveola assembly, are highly expressed in bladder cancer and possibly contribute to the progression of this type of cancer [134].

Moreover, for some cancers where CAV1 is highly expressed, such as pancreatic cancer, the tumor cells are highly sensitive to albumin-bound or conjugated chemotherapeutic drugs [27]. This observation led the authors to hypothesize that CAV1 may be crucial for drug uptake and responsiveness. Indeed, CAV1 overexpression enhanced sensitivity to the drugs and, as expected, downregulation of CAV1 rendered these cells resistant to apoptosis induced by the albumin-conjugated chemotherapeutic agent [27]. Another report focused on the role of CAV1 in the treatment of human EGFR2 (HER2)-positive breast cancers. Specifically, Chao and colleagues observed high levels of CAV1 in this type of cancer and noted that sensitivity to treatment with an antibody–drug conjugate, known as trastuzumab emtansine or T-DM1, mainly depended on the vesicle-trafficking properties of the tumor cells. Specifically, these authors showed that CAV1 colocalizes with the drug in SKBR-3 breast cancer cells that express moderate levels of the protein. Moreover, SKBR-3 cells were at least five times more sensitive than BT-474 cells, which lack CAV1 [31]. Belting and collaborators validated these results by confirming the role of CAV1 in trastuzumab internalization *via* endocytosis. Furthermore, they demonstrated that hypoxia regulates CAV1 redistribution facilitating the translocation of CAV1 from intracellular pools to the plasma membrane, inhibiting trastuzumab internalization and thereby promoting resistance to T-DM1 treatment [17, 69]. These observations suggest that CAV1 may represent an effective prognostic marker for the outcome of T-DM1-treated patients.

In addition, CAV1 regulates other processes modulated by endocytosis and *vice versa* [9, 151]. Such is the case for autophagy, a “self-eating” process used by eukaryotic cells to eliminate unnecessary proteins and organelles *via* the lysosomal pathway and thereby aid in maintaining metabolic homeostasis [142]. Indeed, downregulation of CAV1 promotes autophagy along with lysosome function by membrane raft disruption in breast cancer cell lines and in tumor-compromised tissue [151], suggesting that CAV1 may inhibit breast cancer development by modulating autophagy. Alternatively, others found that the presence of CAV1 in highly metastatic hepatocellular carcinoma cell lines not only promotes cell proliferation, angiogenesis, and migration but also inhibits autophagy [92]. Thus, CAV1 is considered a marker of poor prognosis in hepatocellular carcinoma patients who had undergone tumor resection. The authors suggest that targeted therapy against CAV1 should aid in re-establishing autophagy and thereby serve to treat this type of cancer [92].

## Non-canonical role of CAV1 in peroxisomes

During peroxisome biosynthesis, the peroxins (PEX) 3 and 14 are selectively released from the mitochondria in vesicular structures called pre-peroxisomes. Subsequently, ER-derived vesicles and pre-peroxisomes merge, incorporating peroxisome-forming proteins, such as PEX16, into the structure, leading to increased import competence and peroxisomal maturation [159]. During the elongation and fission processes, PEX11 indirectly recruits dynamin-like protein 1 (DLP1, equivalent to DRP1 [183]) [87]. In fact, PEX11 interacts with the mitochondrial fission factor (Mff) [75], an essential protein for mitochondrial recruitment of DLP1 during mitochondrial fission [120]. Once DLP1 has been recruited to peroxisomes, this protein self-activates and polymerizes, thereby allowing the pinching-off of daughter peroxisomes [75]. Moreover, the silencing of DLP1 or Mff inhibits peroxisomal fission, which leads to the development of tubular peroxisomes [46], suggesting that these proteins participate not only in mitochondrial but also in peroxisomal fission.

In the liver, peroxisomes participate in the β-oxidation of long-chain fatty acids [80] and in the bile acid synthesis through the conversion of cholestanoic acid to cholic acid [126]. Furthermore, peroxisomes are essential for the synthesis of plasmalogens [169], which are required for membrane biogenesis and protection against reactive oxidative species [187]. Interestingly, progression of several tumors correlates with overexpression of peroxisomal enzymes [22], like alpha-methylacyl-CoA racemase, a mitochondrial and peroxisomal enzyme involved in the metabolism of branched-chain fatty acids and bile acid intermediates [154, 185]. On the other hand, peroxisome disruption or inhibition leads to metabolic stress, cancer cell death, and tumor reduction [22].

Information regarding the role of peroxisomal CAV1 is scarce in the literature. However, CAV1 is reportedly enriched in peroxisomal fractions of hepatocytes, specifically in detergent-resistant microdomains [180]. In this sense, CAV1 colocalizes with peroxisomal proteins, such as catalase, 70 kDa peroxisomal membrane protein (PMP70), adrenoleukodystrophy protein (ALDP), PEX14, and bile acid-coenzyme A:amino acid *N*-acyltransferase (BAAT) [180]. In addition, Faber and colleagues reported that, although CAV1 is not essential for peroxisome biogenesis, peroxisomal CAV1 is involved in hepatocyte proliferation and lipid metabolism [180], and aberrant expression of the protein could promote cancer development.

As mentioned previously, CAV1 impairs DLP-1 activation by PKA at the mitochondrial level in cancer cells, which generates mitochondrial instability and dysfunction [19]. Taken together, these data demonstrate the existence of a connection between peroxisomes and mitochondria, not only in terms of their function but also in their maturation. Bearing this in mind, CAV1 presence or absence there may be associated with organelle dysregulation and the development of cancer.

## Non-canonical role of CAV1 in lipid-enriched particles and vesicles

Beyond the literature discussed so far ascribing CAV1 roles in different organelles within the cell, the protein reportedly also exists in a secretable form that may modulate cell function in a paracrine or autocrine manner. Given the characteristics of the protein, several criteria must be met for this to occur. For instance, Anderson and co-workers suggested that the transmembrane and hydrophobic region of CAV1 needed to be embedded in lipid-enriched particles, while the cytoplasmic regions of the protein should face the aqueous phase [86, 90]. Others showed that CAV1 accumulated specifically in the lumen of secretory vesicles, together with apolipoproteins, when pancreatic acinar cells or CAV1-transfected exocrine cells were exposed to secretagogues, such as secretin, cholecystokinin, or dexamethasone [90]. Also, mechanistic insight as to how CAV1 distribution may be modulated came by showing that CAV1 phosphorylation on specific residues, such as serine 80, targeted CAV1 to the secretory pathway rather than to caveolae in AR42J pancreatic adenocarcinoma cells [145].

The physiological role of CAV1 embedded in lipid particles was initially considered relevant in the context of lipid transport between cellular compartments, such as lipid droplets, caveolae, mitochondria, or the ER [86]. However, the notion that secretable forms of CAV1 may be important in the context of cancer arose when Thompson and collaborators showed that androgen-insensitive prostate cancer cells secrete CAV1 and, most importantly, that elevated levels of serum CAV1 were detectable in samples from patients with advanced prostate cancer compared to the levels detected in the serum of healthy subjects [162]. Moreover, these authors evaluated the biological effects of the conditioned media obtained from CAV1-expressing and secreting prostate cancer cells (LNCaP-CAV1) on recipient LNCaP cells lacking CAV1. Following incubation with conditioned media (CM), recipient cells increased their viability and anchorage-independent growth, and these effects were not observed when the CM were depleted with a CAV1-specific antibody. Importantly in an *in vivo* model, consecutive injections of this antibody ablated tumor growth and metastasis of CAV1-expressing mouse prostate cancer cells [162]. In agreement with the latter, Liu and colleagues analyzed the CM obtained from LNCaP-CAV1 prostate cancer cells and found that CAV1 was associated with small lipoprotein particles of approximately 15 to 30 nm in size, suggesting that these particles may promote tumor growth and metastasis *in vivo* [10]. Furthermore, others also detected CAV1 in the CM of human melanoma cells. Specifically, recipient cells exposed to CM increased their migration and invasion capabilities, but such effects were diminished when CM were depleted with a CAV1-specific antibody [40]. Interestingly, Notario and colleagues found that Ewing’s sarcoma cells may regulate their proliferation in an autocrine manner by taking up their own secreted CAV1 [148].

Taken together, the evidence provided so far identified CAV1 as a secretable protein present in small lipoprotein structures that favor tumor development. However, others then identified CAV1, not only in small lipoparticles [10], as mentioned, but also in membrane-enclosed structures called prostasomes. These vesicles, released by metastatic prostate cancer cell lines, such as PC-3, are enriched in membrane raft components and may be several times larger in size than the aforementioned lipoparticles [93]. Such membrane-enclosed structures come in many flavors and are frequently employed as vectors of communication among mammalian cells and currently referred to as extracellular vesicles (EVs). In general, EVs are characterized as containing a heterogeneous mixture of sphingolipids and cholesterol in their membrane bilayer and a large variety of molecular cargos (nucleic acids, lipids, and/or proteins) that may be taken up by nearby or distant recipient cells [102, 171].

Two major types of EVs, which mainly differ in their biogenesis, are considered in the literature. In the case of the aforementioned prostasomes, the vesicles are also known as microvesicles of tumor origin. They originate directly from the plasma membrane by budding and vary in size, ranging from 50 up to 1000 nm in diameter. The second type of EVs, known as exosomes, are much smaller (50–150 nm) in size than the aforementioned microvesicles and have attracted great interest in the area of cancer research due to their widely described role in promoting this process [72, 171]. Exosome genesis begins at the plasma membrane, but the vesicles then undergo a series of modifications at the early and then the late endosome (multivesicular bodies, MVBs) stage. The sorting of molecules into these MVBs takes place by at least two main mechanisms. These may depend on the participation of the endosomal sorting complexes required for transport (ESCRT) machinery and may specifically include syntenin and syndecans. On the other hand, exosome biogenesis has also been described to be regulated by ESCRT-independent pathways, which involve the participation of tetraspanins, lipids, and RabGTPases [1, 62]. Subsequently, vesicles pinch off towards the lumen of MVBs to generate intraluminal vesicles that contain the pre-sorted information and then, upon fusion of the MVB compartment with the plasma membrane, these vesicles are liberated into the extracellular space [102, 171]. Importantly, in the context of cancer, EVs have been assigned highly diverse roles ranging from regulation of immune responses [34] and the tumor microenvironment [130, 153] to preparation of the metastatic niche [72].

A currently unresolved question is how the presence of CAV1 contributes to these processes attributed to EVs. There is evidence that acidic pH conditions favor the delivery of CAV1 in exosomes to less aggressive melanoma cells lacking CAV1. This implies that specific pH conditions may facilitate cell-to-cell communication *via* exosomes, thereby promoting the exchange of specific molecules including CAV1, that modify the recipient phenotype, generally augmenting their tumorigenic properties [122]. Notably, CAV1 was also detected together with other tumor markers in exosomes from plasma samples obtained from melanoma patients. Notably, the ratio of CAV1-containing exosomes diminished significantly when these melanoma patients underwent chemotherapy compared to patients that did not receive such treatment [96]. Furthermore, CAV1 has been detected in EVs from other types of cancer models, apart from melanoma and prostate cancer mentioned so far. For instance, Wong and colleagues showed that only exosomes from metastatic hepatocarcinoma (HCC) cell lines contain CAV1 along with other tumorigenic proteins and RNAs, while this was not the case for exosomes obtained from non-metastatic or immortalized hepatocyte cell lines. Interestingly, the authors showed that the incubation of non-motile HCC cell lines with exosomes derived from motile HCC cell lines leads to an increase in the migration and invasion of these cells and that this may be due to the observed activation of PI3K/Akt and MAPK signaling pathways, as well as the increased secretion of the metalloproteinases MMP-2 and MMP-9 by recipient cells [60].

Evidence from our laboratory also described the possible role of CAV1 in EVs derived from the metastatic human breast cancer cell line MDA-MB-231. Specifically, when analyzing the protein content of these EVs by mass spectrometry, several proteins associated with the biological function “adhesion,” and hence relevant in metastasis, were identified, including tenascin, cysteine-rich angiogenic inducer 61 (Cyr61), and S100 proteins [23]. Importantly, these proteins were only detected in CAV1-containing EVs. Also, *in vitro* exposure of breast cancer cells lacking CAV1 to EVs containing CAV1 increased their migration and invasiveness as compared to recipient cells of EVs derived from MDA-MB-231 cells in which CAV1 was silenced. These results not only indicate that CAV1-containing EVs are capable of transferring malignant traits to recipient cells but also underscore the importance of CAV1 in this process [23]. An intriguing interpretation of this data is that CAV1 plays an important role in defining the molecular cargo of such EVs, a point which needs to be addressed in future studies [24].

All together, these data identify a role for CAV1 in the formation of EVs that specifically favor tumor development, progression, and conditioning of the metastatic niche, although the mechanisms by which this occurs remain to be determined. Also, such studies open up the possibility of developing therapeutic strategies to prevent tumor progression and metastasis by downregulating CAV1 and thereby reducing the transport of pro-metastatic cargoes to nearby and distant recipient cancer cells. Alternatively, and somewhat counter intuitively, CAV1-containing EVs may also be useful in anti-tumor therapies, by aiding in transporting specific cargoes, such as therapeutic drugs and/or nanoparticles, to malignant cells. In this respect, a recent study showed that B16F10 murine melanoma cells incubated with gold nanoparticles (AuNPs) produce exosomes loaded with AuNPs that preferentially accumulate in metastatic lung nodules formed following intravenous injection of these cells [79]. To what extent the presence of CAV1 in EVs may aid in drug delivery is an intriguing but as yet unresolved question.

## Conclusions

**Fig. 2:**
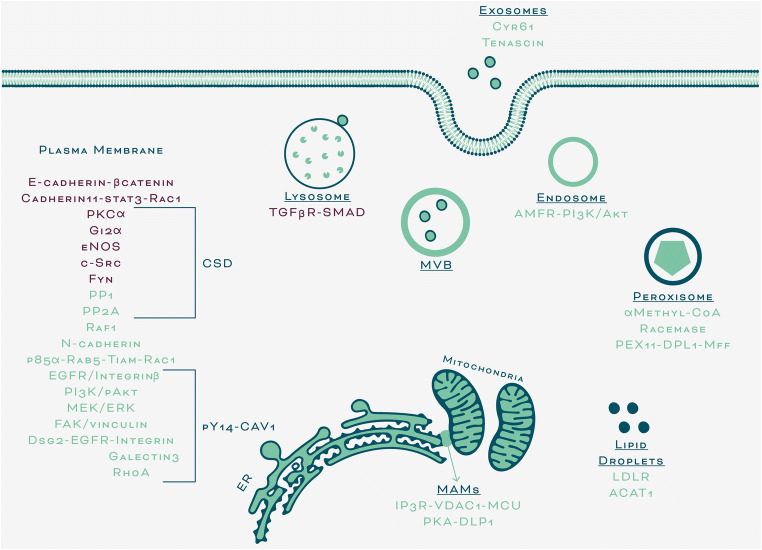
Signaling events modulated by CAV1 at the plasma membrane (canonical role) and at several subcellular localizations (non-canonical role). Proteins regulated by CAV1 functioning as a tumor suppressor are indicated in purple. Alternatively, those proteins modulated by CAV1 functioning as a tumor promoter are shown in green. CSD, caveolin-1 scaffolding domain; pY14-CAV1, phosphorylated caveolin-1; MVB, multivesicular body; MAMs, mitochondria-associated membranes. For protein abbreviations and more details, see the respective sections of the main text

In summary, we focused this discussion initially on literature dealing with what we defined as canonical roles of CAV1, as a modulator of signaling events at the plasma membrane. There, CAV1 is largely considered a scaffolding protein that functions within caveolae; however, while roles for the non-caveolar protein in regulating signaling *via* phosphorylation on tyrosine-14 or modifying plasma membrane lipid composition have been described, they need to be explored in greater detail in the future. Furthermore, we now know that CAV1 expression alters mitochondrial function in a number of ways, yet the relevance of these mechanisms in cancer is still poorly understood, as is also the case for peroxisomal CAV1. The importance of CAV1 in lipid transport is well-established, but again more research on the regulation of these processes and their relevance to tumor biology is necessary. Thus, as stated at the onset, CAV1 is present beyond the plasma membrane in numerous subcellular compartments. However, the precise function of CAV1 at these sites often still remains to be defined, as does the role of these pools in processes related to the development of cancer (non-canonical roles, Fig. 2). Undoubtedly, more research in this area is required to shed light on these emerging new functions of the protein. Finally, understanding how CAV1 participates in EV genesis and function is an exciting new field of CAV1 research that holds considerable promise in the development of cancer therapies.
